# An antibiotic, heavy metal resistant and halotolerant *Bacillus cereus *SIU1 and its thermoalkaline protease

**DOI:** 10.1186/1475-2859-9-59

**Published:** 2010-07-21

**Authors:** Sanjay K Singh, Vinayak R Tripathi, Rakesh K Jain, Surendra Vikram, Satyendra K Garg

**Affiliations:** 1 Department of Microbiology, Dr. Ram Manohar Lohia Avadh University, Faizabad-224001, UP, India; 2 Institute of Microbial Technology, Sector 39 A, Chandigarh-160036, India

## Abstract

**Background:**

Many workers have reported halotolerant bacteria from saline conditions capable of protease production. However, antibiotic resistance and heavy metal tolerance pattern of such organisms is not documented very well. Similarly, only a few researchers have reported the pattern of pH change of fermentation medium during the course of protease production. In this study, we have isolated a halotolerant *Bacillus cereus *SIU1 strain from a non-saline environment and studied its antibiotic and heavy metal resistance pattern. The isolate produces a thermoalkaline protease and changes the medium pH during the course of fermentation. Thermostability of protease was also studied for 30 min.

**Results:**

Seventy bacterial strains isolated from the soils of Eastern Uttar Pradesh, India were screened for protease production. All of them exhibited protease activity. However, 40% bacterial isolates were found good protease producers as observed by caseinolytic zones on milk agar plates. Among them, culture S-4 was adjudged as the best protease producer, and was identified as *Bacillus cereus *by morphological, biochemical and 16 S rDNA sequence analyses. The isolate was resistant to heavy metals (As^2+^, Pb^2+^, Cs^1+^) and antibiotics (penicillin, lincomycin, cloxacillin, pefloxacin). Its growth behavior and protease production was studied at 45°C and pH 9.0. The protease units of 88 ml^-1 ^were noted in unoptimized modified glucose yeast extract (GYE) medium during early stationary phase at 20 h incubation period. The enzyme was stable in the temperature range of 35°-55°C.

**Conclusions:**

An antibiotic and heavy metal resistant, halotolerant *Bacillus cereus *isolate is capable of producing thermoalkaline protease, which is active and stable at pH 9.0 and 35°-55°C. This isolate may be useful in several industrial applications owing to its halotolerance and antibiotic and heavy metal resistance characteristics.

## Background

In modern times, the products of biological origin, particularly enzymes, are attracting the attention of researchers. Their role in several biological and commercial processes has been duly emphasized. Among all the enzymes, proteases occupy an important niche as they were the first to be produced in bulk, and now constitute ~66% of total enzymes employed [1].

Proteases are present in all living organisms, but microbial proteases are most exploited group of industrial enzymes. Based on their mode of action, they are further classified into four categories viz. alkaline, acid, thiol and metallo proteases [2]. Since alkaline (serine) proteases are active over a broad pH (7-12) and temperature (35°-80°C) ranges [3], they are world wide center of attraction for researchers. Several fungi, actinomycetes and bacteria are endowed with the capacity to produce alkaline serine proteases in diverse environmental and agroclimatic conditions [4]. However, bacterial proteases are preferred as they grow rapidly, need less space, can be easily maintained and are accessible for genetic manipulations.

The important protease producing bacteria are species of *Bacillus, Pseudomonas, Halomonas, Arthrobacter *and *Serratia*. Among all bacterial species, bacilli play an important role in production of alkaline protease owing to their chemoorganotrophic nature [5]. Several species of *Bacillus *are industrially employed to produce thermostable alkaline protease as they grow easily under extreme pH and temperature conditions [6]. The enhancement of protease production by genetic manipulation has been well studied in *B. cereus, B. subtilis, B. stearothermophilus*, etc. by a number of researchers, which further underlines the significance of this enzyme [3].

Proteases have diverse applications, mainly in the detergent, food, leather and pharmaceutical industries [3]. Highly thermoalkaline proteases appear to have better washing properties, and if fortified in detergents, the washing can be conveniently performed at 50°-60°C [4]. Recently, microbial proteases have also been employed in the treatment of waste water contaminated with heavy metals and organic matter. Qiuhong et al. [7] have reported a serine protease of *Bacillus *sp. B16 with nematicidal properties.

Keeping the above in view, the present study was envisaged with the following objectives: 1) isolation and screening of thermoalkaline protease producing isolates in search of an efficient strain, 2) its characterization employing morphological and biochemical methods, followed by 16 S rDNA sequence analysis, 3) exploration of antibiotic and heavy metal resistance pattern to elucidate potential of strain under stress conditions, 4) its growth behavior and protease production under unoptimized conditions and 5) study of thermostability of alkaline protease.

## Results and Discussion

### Isolation and screening of thermoalkaline protease producing bacterial cultures

The soil of Eastern Uttar Pradesh region is slightly alkaline, which supports rich and diverse microflora. Seventy bacterial isolates producing variable caseinolytic zones on milk agar plates were isolated from the soil samples. The zones of clearance by isolates reflect their extent of proteolytic activity. Those having clearance zone greater than 3.0 mm were considered as significant [8]. Among 70 bacterial isolates, 28 (40%) exhibited good protease activity which was reassessed by loading their culture broth in the wells on milk agar plates (pH 9). The culture broth of good protease producers cleared more than 3.0 mm zone within 4 h of incubation at 45° ± 1°C, thereby indicating an extra-cellular nature of the protease. The isolate S-4, showing maximum clearance zone diameter was selected for further studies.

### Morphological, biochemical and molecular characterization of isolate S-4

**Figure 1:**
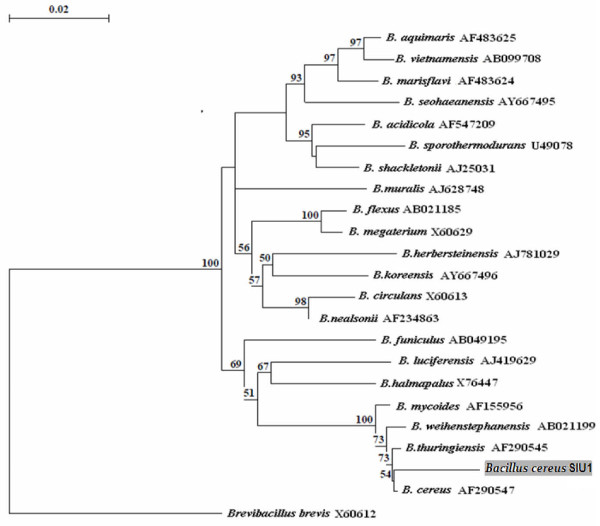
**Phylogenetic tree showing relation between Strain SIU1 and other *Bacillus *strains**.

The isolate S-4 was rod-shaped, Gram-positive, strict aerobe, motile, endospore former (single central spore) with positive catalase and oxidase activity. It grew over a wide range of pH (5-12), temperature (15°-55°C), NaCl concentration (0.0-10%), and was able to hydrolyze casein and gelatin. The strain was halotolerant as it grew in the presence of 0.0-10% NaCl, but did not require salt for its physiological activities. On account of morphological and biochemical characteristics, it was identified as *Bacillus *sp. The isolate was deposited to MTCC (accession number MTCC 9778), Institute of Microbial Technology, Chandigarh (India). Analysis of 16 S rDNA sequence (1456 bp) revealed its 99% homology with *Bacillus cereus *strains, and was designated as *Bacillus cereus *SIU1. The 1456 bp 16 S rDNA sequence was submitted to Genbank [FJ:976896]. The strain SIU1 was in the same cluster of phylogenetic tree (Fig. 1) with different strains of *B. cereus*. However, the 16 S rDNA sequence analysis indicates that it is a different and novel strain of *B. cereus*. The 16 S rDNA is the most widely accepted gene employed for bacterial classification and identification. Goto et al. [9] suggested that 5' end region (~275 bp) is the hypervariant (HV) region in the gene, highly specific for each type strain, and considered as an useful index for identification or grouping of *Bacillus *sp. Gupta et al. [1] emphasized that use of molecular markers like 16 S rDNA as species-specific identification tool have provided with a truly "microscopic" sensitivity down to single-cell detection.

### Antibiotic sensitivity assay and heavy metal resistance pattern

The strain SIU1 was resistant against penicillin, lincomycin, cloxacillin and pefloxacin, while sensitive to other antibiotics tested. Antibiotics, for which the isolate is resistant, may be supplemented to fermentation medium during enzyme production so as to check the contamination by other sensitive isolates. The organism also exhibited a high degree of tolerance to elevated concentrations (μg ml^-1^) of lead (600), arsenic (2300) and cesium (2100). It was tolerant to fairly high concentrations of cobalt (225), nickel (175) and chromium (125) as well, but sensitive to even very low concentration of selenium (25) and mercury (20). In leather industries, this isolate may be useful for dehairing process as the use of chromium is very common in the tanning process [10]. Further, the protease producing organisms displaying heavy metal tolerance may be of potential use for the treatment of multimetal contaminated sludge generated during wastewater treatment.

The increased load of antibiotics/disinfectants in health care and heavy metals in industries creates the selective pressure for the survival of bacteria in a contaminated environment. Thus, in a multiple stressed environment, bacterial cells acquire resistance/tolerances by alterations in genetic makeup either by mutation or transfer of resistant genes among the bacteria.

### Culture conditions and medium selection

**Figure 2:**
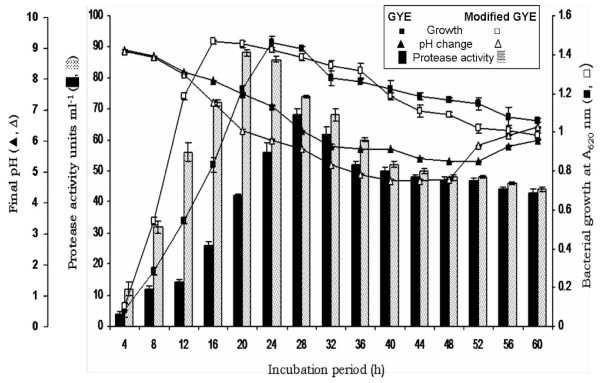
**Growth behavior of *Bacillus cereus *SIU1, pH change and production of extracellular protease in GYE and modified GYE media at initial pH 9.0, 45°C and 120 rpm during 60 h growth**.

The SIU1 strain exhibited typical sigmoidal growth behavior in both the culture media. In glucose yeast extract (GYE) broth, stationary phase commenced at 24^th ^h, while in modified GYE broth, the onset of stationary phase was at 14^th ^h onward after a steep log phase. In both the media, the stationary phase of bacterial growth witnessed maximum protease production. However, the alkaline protease production was maximum (88 Uml^-1^, 20.95 U/mg) in modified GYE medium at 20 h, while it was 68 Uml^-1 ^(11.72 U/mg) at 28 h in GYE medium (Fig. 2). Hence, modified GYE medium was selected for further studies on protease production.

Production of protease during the stationary phase of growth is in accordance with the studies of other workers [5,7,11,12]. Maximum protease production during stationary phase may be due to high rate of protein turnover for the sporulation process. The formation of endospores in bacteria involves extensive protein turnover, required for the synthesis of spore specific proteins and enzymes [13]. Proteases are well known to participate in proper protein turnover of cell. Evidence for participation of proteolytic activity in regulation of protein turnover was demonstrated by lack of proper turnover in protease-deficient mutants [3]. The requirement of protease for bacterial sporulation has also been demonstrated by use of protease inhibitors [14]. Zucca and Balassa [15] have also found that *Bacillus subtilis *produces two extracellular proteases during sporulation stage.

The pH of modified GYE medium started declining gradually after 4 h of growth from 9.0 to 4.71 up to 48 h, and thereafter increased to 6.4 at 60 h (Fig. 2). The drop in pH may be attributed to the production of organic acids, which were consumed during later stages of growth resulting in slight pH increase to 6.4. Our findings are in accordance with Abusham et al. [16] who have reported similar trend in pH change of *Bacillus subtilis *strain Rand culture medium during 48 h growth albeit at different time intervals. In our study, the growth and protease production was detected at as early as 4 h (pH ~8.8) and reached maximum at 20 h of incubation (final pH ~6.3). Hence, irrespective of decrease in pH, the protease was produced during broad pH change from 8.8 to 4.71. Further, the bacterial growth as well as protease production was inversely related to decrease in pH of modified GYE broth during 20 h incubation. However, at an extreme acidity (final pH 4.71), the production of protease was greatly reduced at 48 h (Fig. 2). The protease production maxima at slightly acidic pH (6.3) is in agreement with the findings of Gouda [11] who has reported final pH in slightly acidic to slightly alkaline range for maximum protease production by *Bacillus *sp. MIG during 72 h incubation. Abusham et al. [16] have also reported maximum protease production at final pH drop nearly to 6.5 by *Bacillus subtilis *strain Rand at 20 h growth. Our results on growth behavior of *Bacillus cereus *SIU1 isolate and protease production with respect to pH change are significant in the sense that when the initial pH of modified GYE medium was maintained throughout at 9.0 or 6.0, both the growth and protease production were greatly reduced (data not shown). Thus, we may infer from our results that initial alkaline pH 9.0 is essential for induction of alkaline protease production. It requires further gradual fall to slightly acidic pH of 6.3 for maximum enzyme production. The pH of culture broth strongly affects enzymatic processes and transport of compounds across the cell membrane. However, the molecular basis of pH affecting bacterial metabolism in culture broth is obscure. Since proton motive force in chemiosmosis is affected by the medium pH value, it is possible that under optimum pH range, the relative metabolic efficiency is high.

The temperature (45°C) for protease production is in agreement with other workers, reporting enzyme production in a wide temperature range of 37°-80°C [5,13,17]. Higher enzyme units in less time in modified GYE than GYE broth was supposedly due to supplementation of peptone in the medium. It has already been demonstrated by other researchers that addition of complex organic nitrogen source(s) stimulates the protease production [17].

### Thermostability of protease

**Figure 3:**
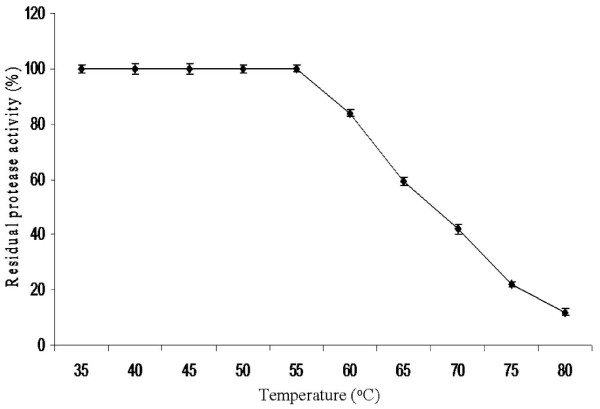
**Thermostability of alkaline protease of *Bacillus cereus *SIU1 after heating for 30 min at 35°-80°C**.

The alkaline protease of strain SIU1 was completely stable in the broad temperature range of 35°-55°C during 30 min incubation. However, with further increase in every 5°C temperature, there was a gradual decrease in enzyme stability ranging between 10-24% upto 80°C. The enzyme retained 84, 60 and 42% activity even after treatment at 60, 65 and 70°C, respectively. Even at 80°C, residual protease activity was found 12% after 30 minute heating (Fig. 3). The protease of strain SIU1 is more thermostable than proteases studied by several other researchers. Abusham et al. [16] have reported a thermostable protease stable up to 55°C but retained only ~40% activity at 65°C. A thermostable protease of *Pseudomonas aeruginosa *PseA retained 80% of its initial activity after 30 min heating at 55°C [17]. Two different proteases Pro 1 and Pro 2 of *Bacillus *sp. MIG retained ~85-90% activity after 30 min heating at 50°C [11]. Hence it is evident that the alkaline protease of strain SIU1 is more thermostable, and may be applied to several biotechnological and industrial purposes.

## Conclusions

A thermoalkaline protease is produced by a novel isolate *Bacillus cereus *SIU1. The organism appears to have greater potential for enhanced enzyme production through optimization of nutritional and physical parameters. Resistance against heavy metals and antibiotics facilitates its use for various processes under stressed conditions. Owing to its halotolerant thermoalkaline nature, its protease may have potential uses in industries such as detergent, food, pharmaceutical, leather, agriculture, etc. as well as molecular biology techniques. Further studies on biochemical and structural characteristics of protease are under investigation.

## Methods

### Isolation and screening of thermoalkaline protease producing bacteria

The soil samples were collected aseptically from different districts of Eastern Uttar Pradesh, India to isolate protease producing bacteria. One g soil was suspended in 9.0 ml sterile distilled water, agitated for a min and 0.1 ml suspension was spread over milk agar plates (pH 7) containing (gl^-1 ^distilled water): skimmed milk, 20.0; agar, 15.0, and incubated for 20-30 h at 37° ± 1°C. Bacterial colonies showing clear zones were selected, streaked twice on milk agar plates for purification and maintained as pure culture over nutrient agar slants (pH 7, 4°C). Bacterial isolates having maximum protease activity, as measured by caseinolytic zone diameter (mm) [8], were studied for protease production in glucose yeast extract (GYE) broth (pH 9) containing (gl^-1 ^distilled water): glucose, 15.0; yeast extract, 5.0 and CaCl_2_.2H_2_O, 0.2 [12]. One hundred μl of 24 h culture broth of each isolate was loaded in the wells created on milk agar plates (pH 9) and incubated for 4 h at 45° ± 1°C. The isolate having maximum clearance zone was selected for further studies.

### Morphological, biochemical and molecular characterization of selected isolate

The selected bacterial isolate S-4 was identified by morphological and biochemical characterization as per the Bergey's Manual of Systematic Bacteriology [18]. Molecular characterization was done by 16 S rDNA sequence analysis. Bacterial genomic DNA was extracted using Axygen Genomic DNA extraction Kit, and PCR amplification was employed with 16 S universal primers: 27F (5'-AGAGTTTGATCCTGGCTCAG-3') and 1492R (5'-TACGGTTACCTTGTTACGACTT-3') at annealing temperature of 50°C (25 cycles). The DNA fragment of ~1.4 kb was eluted from gel using Qiagen gel extraction kit and employed as template for amplification of forward and reverse strands using 27F and 1492R primers, respectively in separate sequencing PCR tubes. The sequencing was carried out using ABI Prism-310 automated sequencer. The sequence of closely related taxa of the isolate was retrieved from the GenBank database using http://www.ncbi.nlm.nih.gov/BLAST[19]. The phylogenetic tree was constructed by neighbor joining (NJ) method; the significance of junctions was established using bootstrap method (1000 replicates).

### Antibiotic sensitivity assay and heavy metal resistance pattern

The antibiotic sensitivity pattern of selected isolate was studied by disc diffusion method [20]. The antibiotics (μg/disc) used were cephalexin (30), penicillin (10), kanamycin (30), co-trimoxazole (25), ampicillin (10), amoxycillin (10), tetracycline (30), erythromycin (15), lincomycin (15), cloxacillin (5), amikacin (30), cefaclore (30), cefuroxime (30), ceftriaxone (30), ofloxacin (5), cefadroxil (30), ceftaxidime (30), cefotaxime (30), pefloxacin (5), cefazoline (30), ciprofloxacin (5) and norfloxacin (10). Antibiotic impregnated discs were placed over freshly prepared bacterial lawn on Mueller Hinton agar (HiMedia Laboratories Pvt. Ltd. India) plates and incubated at 35° ± 1°C for 24 h. The isolate was classified as resistant or sensitive by the presence/absence of inhibition zone of growth around antibiotic discs.

To determine the heavy metal resistance pattern, 0.1 ml of bacterial culture having 0.5 OD (A_620_; 1 cm cuvette) was spread aseptically on Mueller Hinton agar plates, supplemented with different concentrations (μg ml^-1^) of the following heavy metals: Pb (0.0-700), As (0.0-2500), Cr (0.0-200), Ce (0.0-2500), Hg (0.0-50), Se (0.0-50), Ni (0.0-250) and Co (0.0-300). The metal salts used were lead acetate [Pb(CH_3_COO)_2_], sodium arsenate [Na_2_HAsO_4_], potassium dichromate [K_2_Cr_2_O_7_], cesium chloride [CsCl], mercuric chloride [HgCl_2_], selenium sulphide [SeS], nickel chloride [NiCl_2_] and cobaltous chloride [CoCl_2_]. Bacterial growth was observed during 24-48 h at 35° ± 1°C.

### Culture conditions and medium selection

The selected isolate designated as SIU1 was grown in glucose yeast extract (GYE) and modified (in our laboratory) GYE broth. The modified GYE broth contained (gl^-1 ^distilled water): glucose, 10.0; peptone, 10.0; yeast extract, 5.0 and NaCl, 5.0. To study the growth behavior and protease production, 1.0 ml of mother culture having 0.5 OD (A_620_; 1 cm cuvette) containing 3.4 × 10^7 ^cfu ml^-1 ^was inoculated in 99 ml of broth (pH 9; adjusted after autoclaving using sterilized 1 M Na_2_CO_3_) in Erlenmeyer flasks and incubated at 45° ± 1°C on incubator shaker (120 rpm) for 60 h. At 4 h interval, bacterial growth was assessed by turbidity measurement at 620 nm. Each sample was centrifuged at 16,000 g (4°C) for 5 min and cell-free supernatant assayed for protease activity. The pH change in culture broth was periodically measured during the course of growth.

### Enzyme assay

The proteolytic activity was assayed by casein digestion method of Anson [21]. One ml of enzyme was incubated with 3.0 ml of casein (1% (w/v) in 100 mM sodium carbonate-bicarbonate buffer; pH 9) at 55° ± 1°C. The reaction was stopped after 10 min by addition of 3.0 ml of 10% (w/v) trichloro acetic acid (TCA). The mixture was centrifuged at 16,000 g (4°C) for 10 min, and supernatant used to estimate the amount of free tyrosine as per Lowry et al. [22] using tyrosine as standard. One unit of enzyme activity was defined as the amount of enzyme that liberates 1.0 μg of tyrosine min^-1 ^ml^-1^.

### Thermostability of enzyme

The effect of temperature on the stability of alkaline protease stability was studied. The culture supernatant containing protease was incubated for 30 min at different temperatures in the range of 35°-80°C. The treated enzyme was immediately transferred to 0°C and kept for 15 min before activity measurement. The protease activity was assayed as per the method of Anson [21].

### Statistical analysis

The experiments were performed thrice, each in triplicate. Standard deviation for each experimental result was calculated using Microsoft Excel.

## Competing interests

The authors declare that they have no competing interests.

## Authors' contributions

SKS carried out the research work and drafted the manuscript. VRT was involved in revising the manuscript critically for important intellectual content. RKJ authenticated the data of bacterial identification. SV was involved in data analysis of 16 S rDNA sequencing and preparation of phylogenetic tree. SKG has designed the experiment, contributed substantially to analysis and interpretation of data and has given final approval of the version to be published. All authors read and approved the final manuscript.
